# Improving Clinician Confidence in Insulin Prescriptions at Discharge for Individuals With Newly Diagnosed Type 1 Diabetes

**DOI:** 10.7759/cureus.43643

**Published:** 2023-08-17

**Authors:** Haris Shoaib, Yunus K Hussain, Shahida Ahmed

**Affiliations:** 1 Medicine, Basildon and Thurrock University Hospital, Basildon, GBR; 2 Pediatrics, Basildon and Thurrock University Hospital, Basildon, GBR

**Keywords:** pediatric endocrinology, quality improvement (qi), prescription audit, hospital discharge, multiple daily injection (mdi), type 1 diabetes mellitus (t1dm)

## Abstract

Background

In a questionnaire, we found that pediatric clinicians at Basildon and Thurrock University Hospital (BTUH) have low confidence levels in prescribing multiple daily injections (MDI) for newly diagnosed pediatric patients with type 1 diabetes mellitus. We designed and evaluated locally tailored prescription guidance to improve confidence in MDI discharge prescriptions for pediatric doctors of all grades.

Methods

We designed a prescription guidance tool by adapting existing local guidelines to improve clinician familiarity with MDI prescriptions and prevent prescription errors. The intervention was delivered in a single pediatric unit to doctors of all levels. Feedback was collected, and the clinicians' confidence in their MDI prescriptions was evaluated before and after the intervention. Questionnaires were distributed to all pediatric doctors within the unit to assess their confidence in prescribing MDIs using a five-point Likert Scale. Furthermore, the questionnaires aimed to determine whether clinicians regularly consulted the existing local guidelines. Local guidelines were adapted in consultation with the local pediatric diabetic multidisciplinary team (MDT) and with reference to the East of England Pediatric Diabetes Network to present MDI guidance in a more concise format, which includes an example MDI discharge medication checklist. Following approval by the local guidelines management group, additional changes were made to enhance the practicality and accessibility of the discharge prescription guidance for clinicians. These guidelines were distributed to the pediatric MDT via email and displayed in visible areas of the department.

Results

Out of the 13 doctors surveyed, 10 provided pre- and post-intervention feedback (77%). Statistical significance was calculated using unpaired t-tests. Ninety percent of pediatric doctors routinely refer to local guidelines for guidance on MDI prescriptions. However, 50% of respondents felt that existing local guidelines were not easily accessible, given the time and effort required to locate them. The mean confidence score for completing MDI prescriptions at discharge before the intervention was 1.9 (SD: 0.83). After the intervention, it increased to 4 (SD: 0.63) (95% CI: 2.79-1.41, p<0.0001). Ninety percent of pediatric doctors felt that the design and display of the MDI guidelines optimized patient care.

Conclusions

Following the presentation of the project at a local audit and quality improvement (QI) meeting, the adapted guidelines were included in the junior doctor induction program at BTUH and made accessible on the local intranet. The production of locally tailored prescription guidance for MDI prescriptions at discharge has led to an increase in the confidence of pediatric doctors when writing their prescriptions. We aimed to continue updating this guidance as necessary and making further developments to enhance clinician confidence.

## Introduction

A pediatric unit in the United Kingdom (UK) typically consists of junior doctors at varying stages of their careers, working alongside fully qualified consultants [1]. The junior doctor pediatric team at Basildon and Thurrock University Hospital (BTUH) includes foundation-year doctors (FY1 and FY2), general practice specialty trainees (GPST), and pediatric specialty trainees (ST1-ST8).

The junior doctor team is usually expected to write the majority of medical prescriptions in the hospital, particularly for discharge medications [2]. Within the United Kingdom, it is estimated that junior doctors in their foundation training write approximately two-thirds of all in-hospital prescriptions. Clinically significant prescribing errors are found in approximately 10% of these cases [3].

Insulin-related medication errors have previously been described as "common" throughout the inpatient journey, even at the point of discharge [4]. Despite the measures implemented in 2010 by the National Patient Safety Agency (NPSA) in the United Kingdom to reduce insulin prescription errors, the National Diabetes Inpatient Audit has revealed high levels of errors associated with insulin prescriptions [5,6]. In England, 40% of inpatient hospital drug charts for insulin-treated diabetes contain at least one prescription error related to insulin [6]. An observational study conducted in France examined medication errors among 671 diabetic patients. The study found that individuals with type 1 diabetes were nearly twice as likely to experience medication errors upon admission compared to those with type 2 diabetes. Additionally, 9% of individuals with type 1 diabetes had medication errors at discharge [7].

Junior doctors have been shown to feel inadequately prepared for prescribing medications for diabetes [8]. This is supported by a national online survey, which revealed that junior doctors had reduced confidence in managing all diabetes-related domains compared to other similar areas of medicine [9]. Additionally, junior doctors have reported dissatisfaction with their training in writing prescriptions, with practical prescribing training being deemed suboptimal [10,11].

There is a lack of research in the literature regarding hospital doctors' confidence in prescribing insulin upon discharge. In the context of this study, our aim was to investigate the self-assessed confidence of hospital doctors in writing discharge prescriptions for individuals with newly diagnosed type 1 diabetes. Additionally, we will develop and assess educational guidelines to improve doctor confidence.

## Materials and methods

A prospective, longitudinal, descriptive study was conducted between January 2023 and March 2023 to evaluate clinicians' confidence in prescribing multiple daily injections (MDI) of insulin at discharge. Thirteen doctors participated in this survey, assessing their confidence in MDI prescriptions. Out of these clinicians, 10 met the study protocol inclusion criteria, which required them to be doctors working in the pediatric unit at BTUH from January 2023 to March 2023 and to complete both the pre- and post-intervention questionnaires. Any clinician who did not complete both the pre- and post-intervention questionnaires was excluded from the study.

**Figure 1:**
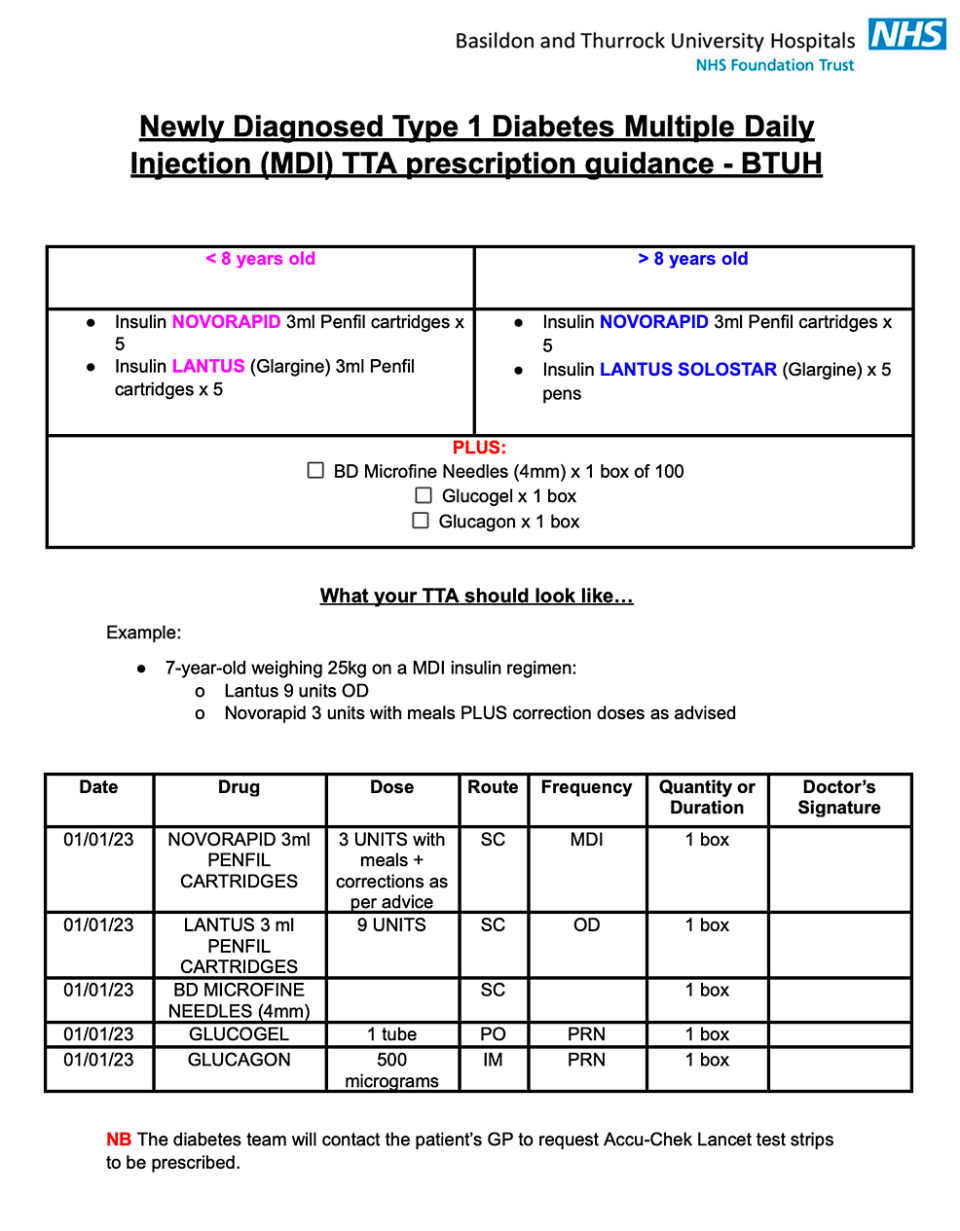
Locally tailored "MDI prescription guidance" introduced to visible areas of pediatric department and sent via email to all members of the pediatric department. TTA: to take away (discharge medication); OD: once daily; SC: subcutaneous; PO: per oral; IM: intramuscular; PRN: pro re nata (as required); GP: general practitioner This figure contains material taken from the East of England (EoE) CYPD Network Newly Diagnosed Care Pathway 2021 (EoE Paediatric Network Shared Guidelines Group; https://www.cypdiabetesnetwork.nhs.uk/east-england/key-documents/) and BTUH local guidelines on the management of type 1 diabetes (https://adfs.mse.nhs.uk/adfs/ls/wia).

Existing local guidelines for multiple daily injections (MDI) of insulin were adapted to increase the confidence of pediatric doctors when prescribing discharge medications for individuals with newly diagnosed diabetes. The guidelines were adapted to be displayed in a more concise format, specifically as an A4-sized poster, to facilitate ease of use for pediatric doctors (Figure 1). These adaptations included a discharge medication checklist for prescribing insulin at discharge for two age groups as follows: children aged above and below eight years. Additionally, the quantities of microfine needles, glucogel, and glucagon required upon discharge were specified. The discharge medication checklist was also based on the criteria for discharge equipment specific to the relevant age groups. To further simplify the task and provide better guidance for clinicians, the poster included an example discharge prescription for an individual who has recently been diagnosed with type 1 diabetes.

Adaptations to the existing guidelines were made after referring to the East of England Pediatric Diabetes Network Shared Guidelines Group and consulting with the local multidisciplinary team for pediatric diabetes which includes specialist nurses and consultants [12]. Prior to distributing the guidelines to the pediatric department, they were reviewed by the local guidelines management group and subsequently granted approval. Following local approval, further revisions were made to the guidelines to ensure that the guidance for insulin discharge medications was more practical and accessible for clinicians to use. These guidelines were then uploaded to the local intranet and displayed in prominent areas of the pediatric department, such as near frequently used computer stations and on poster boards in seminar rooms. To improve accessibility for pediatric trainees, the guidelines were distributed as a PDF file to all members of the pediatric department at BTUH. This allowed them to access the guidelines digitally on their mobile devices or via their email accounts. This has enabled easier access for pediatric doctors, without having to navigate the local intranet or physically locate the displayed posters.

To evaluate the impact of the adapted guidelines, pediatric doctors were requested to complete online questionnaires. These questionnaires were designed and constructed by the authors using Google Forms. They were distributed to the pediatric department via email both before and after the launch of the adapted guidelines. All items in the questionnaire were based on clinical experience, discussions with a pediatric diabetes consultant, and a thorough review of relevant literature. Doctors were asked to rate their overall confidence in prescribing prompt and accurate MDI discharge prescriptions for individuals with newly diagnosed type 1 diabetes as the primary outcome. This was achieved using a five-point Likert scale, ranging from one (not confident at all) to five (very confident). Routine reference to local guidelines, ease of use, and accessibility of guidelines were assessed as secondary outcomes. Opportunities to provide free-text suggestions for improving the project were available in the questionnaires.

For continuous data, characteristics were presented as mean±standard deviation. Categorical data was presented as number (percentage) of participants. Pre- and post-intervention confidence scores were compared using unpaired t-tests, with a statistical significance threshold of p-values <0.01 for the primary outcome. All statistical tests were carried out using GraphPad Prism version 10.0 (Boston, MA: GraphPad Software).

## Results

In total, 13 doctors were surveyed, with 10 doctors completing both pre- and post-intervention questionnaires (Tables 1-3). Of the 10 doctors (77%) who completed both questionnaires, three were FY1s, three were senior house officers (SHOs) in pediatrics, and four were specialty registrars in pediatrics of various grades (ST3-ST8). Of the remaining three participants who completed pre-intervention questionnaires only, one was an FY2, one was a pediatric specialty registrar, and one was a pediatric consultant. All participants who did not complete the post-intervention questionnaire did so due to non-response.

**Table 1:** Pre-intervention questionnaire results (n=10). TTA: to take away (discharge medication); T1D: type 1 diabetes; trust guidelines: existing local guidelines; SHO: senior house officer

Question	Response	n (%)
What is your current grade?	FY1	3 (30%)
	FY2	0 (0%)
	SHO	3 (30%)
	Registrar	4 (40%)
	Consultant	0 (0%)
	Medical student	0 (0%)
	Nurse	0 (0%)
Have you had to prescribe a TTA for a newly diagnosed T1D patient before?	Yes	7 (70%)
	No	3 (30%)
How confident do you feel about prescribing a prompt and accurate TTA for a newly diagnosed T1D patient?	1 (not confident at all)	4 (40%)
	2	3 (30%)
	3	3 (30%)
	4	0 (0%)
	5 (very confident)	0 (0%)
When prescribing a TTA for a newly diagnosed T1D patient, do you have to refer to the trust guidelines for reference?	Yes	9 (90%)
	No	1 (10%)
Do you feel that accessing the trust guidelines for prescribing a TTA for a newly diagnosed T1D patient is easily accessible (consider time and ease of locating the guidelines)?	Yes	5 (50%)
	No	5 (50%)
How beneficial do you think it will be to display posters around the department clearly outlining how to prescribe a TTA for a newly diagnosed T1D patient?	1 (not useful at all)	0 (0%)
	2	0 (0%)
	3	0 (0%)
	4	2 (20%)
	5 (very useful)	8 (80%)

**Table 2:** Free-text suggestions from the pre-intervention questionnaire. TTA: to take away (discharge medication); PT: patient; px: prescription

Question	Response
Do you have any other suggestions for improvement?	“Have an email sent out with the info for TTAs so then we can easily find the info on our emails and have it saved on our phones.”
	“Prescription stickers would be really useful on the ward! So you put the sticker, circle which per age of PT, and then sign and add insulin doses. To ease timely px and reduce error rates.”
	“A discharge checklist will also be helpful.”
	“Ensuring TTAs are prescribed on admission not on discharge as this often delays discharge.”

**Table 3:** Post-intervention questionnaire results (n=10). TTA: to take away (discharge medication); T1D: type 1 diabetes; SHO: senior house officer

Question	Response	n (%)
What is your current grade?	FY1	3 (30%)
	FY2	0 (0%)
	SHO	3 (30%)
	Registrar	4 (40%)
	Consultant	0 (0%)
	Medical student	0 (0%)
	Nurse	0 (0%)
How confident do you feel about prescribing a prompt and accurate TTA for a newly diagnosed T1D patient after posters have been displayed throughout the department?	1 (not confident at all)	0 (0%)
	2	0 (0%)
	3	2 (20%)
	4	6 (60%)
	5 (very confident)	2 (20%)
Are the TTA guidance posters visible and easy to access in the department?	Yes	2 (20%)
	No	8 (80%)
Do you feel that the design and display of TTA guidance posters have helped to optimize patient care?	Yes	9 (90%)
	No	1 (10%)

Seventy percent of the participants had prior experience in prescribing MDI discharge medications. Fifty percent of the surveyed doctors felt that the existing local guidelines for prescribing MDI discharge medications for individuals with newly diagnosed type 1 diabetes were not easily accessible in terms of time and ease of locating the guidelines. Ninety percent of doctors routinely refer to local guidelines as a reference when prescribing MDI discharge medications for individuals with newly diagnosed diabetes. All of the clinicians agreed that displaying posters around the department with discharge prescription guidance would be beneficial. In fact, 80% of doctors rated it as a five on the five-point Likert scale for usefulness.

The mean level of confidence among clinicians in prescribing MDI discharge medications for individuals with newly diagnosed diabetes significantly increased after the introduction of the adapted guidelines (1.9 {SD: 0.83} pre-intervention vs. 4 {SD: 0.63} post-intervention {95% CI: 2.79-1.41, p<0.0001}) (Table 4). Only 20% of the clinicians surveyed found the guidance posters to be visible and easily accessible within the pediatric department. When compared to existing local guidelines, 90% of surveyed doctors felt that the design and display of the adapted guidelines helped optimize patient care.

**Table 4:** Mean clinician pre- and post-intervention confidence scores (n=10). Confidence scores are rated on a five-point Likert scale ranging from one (not confident at all) to five (very confident).

Variables	Minimum	Maximum	Mean	SD
Pre-intervention clinician confidence	1	3	1.9	0.83
Post-intervention clinician confidence	3	5	4	0.63

## Discussion

Adapted guidelines on insulin discharge medications, which include an example prescription and a discharge medication checklist, have increased doctors' confidence in prescribing discharge medications for individuals who have recently been diagnosed with type 1 diabetes. The increased accessibility and ease of use of the guidelines were well-received, with results suggesting that doctors have found the educational intervention to ultimately improve patient outcomes.

While the majority of doctors in this study reported having previous experience in prescribing MDI discharge medications, their confidence levels in writing these prescriptions remained low. With many factors contributing to low confidence and insulin prescription errors, the level of training of the prescriber is a significant factor. This is because the majority of prescriptions are written by junior doctors within the first two years of graduating [3,13]. It is also important to note that this study included clinicians of all grades, who are at different stages of training. Senior clinicians are likely to have greater experience in writing MDI prescriptions than their junior colleagues. While training programs have been implemented nationally to ensure clinicians possess the appropriate knowledge and skill set for prescription tasks, the development of innovative strategies that are sensitive to the local context and increase adherence to prescription guidelines has been associated with a reduction in prescription errors [14]. The demonstrated increase in clinician confidence in discharge prescribing tasks has encouraged adherence to guidelines, thereby optimizing patient care and improving the clinician experience within the pediatric unit.

A strength of the guidelines is that they have been adapted from evidence-based guidelines within the East of England Pediatric Network. Additionally, they have been adapted in consultation with experienced pediatric clinicians and multidisciplinary diabetic team members who are aware of the most common prescription errors. They are able to provide adaptations to the guidelines that offer the greatest benefit to clinicians in order to reduce such errors. The multidisciplinary team was able to design the intervention in a way that would be acceptable for all health disciplines involved in the discharge process, thereby increasing the viability of the educational intervention.

As these guidelines were introduced during a single rotation, and with rotational trainees typically rotating every four months in the United Kingdom, the survey had a low number of participants [15]. However, the results have been very encouraging and support the incorporation of the guidelines into the hospital's pediatric program and network. Self-reported measures have their limitations, as there is a possibility of over-reporting confidence or a lack of correlation between self-reported confidence and proficiency in the clinical task [16]. To mitigate self-report bias, survey participants were provided with anonymous identification. However, the virtual questionnaires and guidance provided ensured that participants were aware of the expectations placed upon them. As a result, they were able to accurately assess their confidence levels, reducing the likelihood of making inappropriate self-assessments.

The surveys were completed by only 10 participants, which is less than half of the doctors in the pediatric unit. This limited sample size hinders the interpretation of the results. Virtual questionnaires were sent to the department via email in an effort to improve accessibility and address the challenges of arranging face-to-face contact caused by varying shift patterns. To enhance participant response rates in future surveys, we will explore strategies to individually contact participants within the hospital setting.

While this educational intervention has focused on the training needs of doctors, there is also a need for allied healthcare professionals working in the management of diabetic care, such as diabetic specialist nurses, dietitians, and staff nurses, to be confident in their ability to manage patients with diabetes [17]. This is to ensure optimal patient outcomes for all individuals newly diagnosed with type 1 diabetes. In the future, we aim to create additional adaptations to this guidance in order to provide more specific interventions for the multidisciplinary team responsible for the discharge preparation of diabetic patients.

Not all discharge prescriptions for individuals with type 1 diabetes will be reviewed by the diabetes pharmacists or diabetes multidisciplinary team, especially when patients are discharged outside of regular working hours. To ensure quality improvement and reduce prescription errors, the guidance should promote the completion of discharge prescriptions upon admission or before the patient's discharge, especially when in-hours services, such as diabetes pharmacists or multidisciplinary teams, are accessible to provide support and validate the prescriptions.

The poor visibility of posters in the pediatric department has been shown to contribute to information overload, particularly when a clinical poster is displayed in non-protected areas or alongside other research posters and guidance [18]. This has been the case with this particular intervention. To promote the accessibility of the intervention and adherence to the guidelines, additional approaches should be considered. These may include introducing prescription stickers with discharge medication checklists to be added to clinical notes or incorporating the intervention into discharge packs on pediatric wards.

Free text comments highlighted the need for further incorporation of the guidance into the diabetic discharge education pack at the hospital. The guidance was later included in the junior doctor induction program after being presented at the local quality improvement (QI) and audit meeting. It is hoped that the guidance will become part of the local integrated care pathway for individuals newly diagnosed with type 1 diabetes.

## Conclusions

Our revised guidelines have increased confidence among pediatric clinicians in prescribing discharge medications for individuals with newly diagnosed type 1 diabetes. The guidelines will serve as a foundation for the development of future educational interventions, taking into consideration the feedback from survey participants. Future development will involve the use of prescription labels to further ease prescription tasks for clinicians. Additionally, the guidance will be included as part of discharge packs for diabetic education to enhance the education provided to patients. With a multidisciplinary approach to our educational intervention, we are keen to expand our efforts in order to improve discharge preparation for individuals newly diagnosed with type 1 diabetes.
